# Supplementary figures and images for: An auditory cortical-striatal circuit supports sound-triggered timing to predict future events
Source: PLoS Biol. 2025 Jun 2;23(6):e3003209. doi: 10.1371/journal.pbio.3003209 (PMC12169527; doi:10.1371/journal.pbio.3003209)

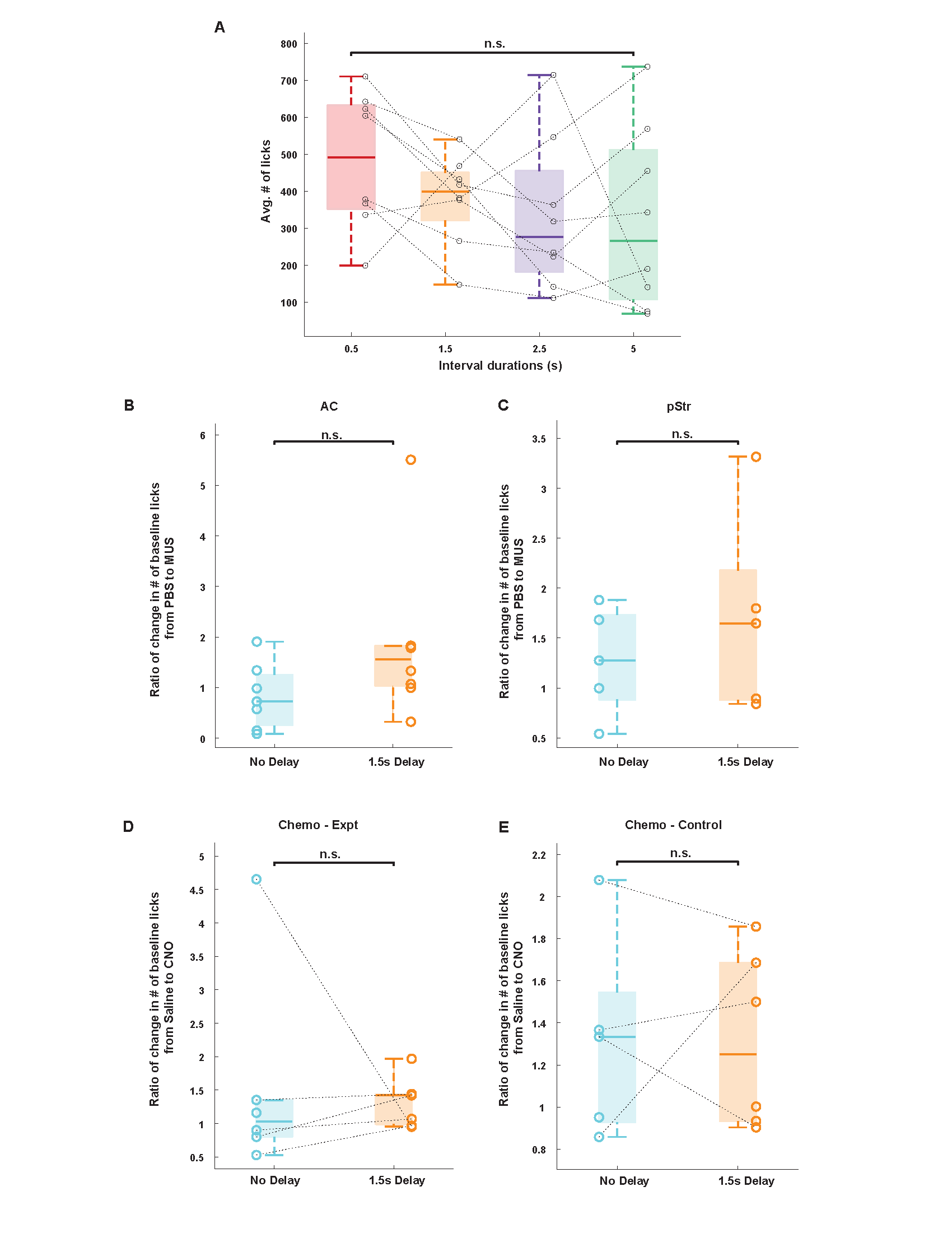

Supplement: S1 Fig — A. Boxplot showing the distribution of licking during the baseline period across animals across sound-reward intervals on the best behavior days are not significantly different (p = 0.251, Kruskal–Wallis test). In each box, the solid line indicates the median, and the bottom and top edges of the box indicate the 25th and 75th percentiles, respectively. The whiskers extend to the minimum and maximum values in the dataset. Individual circles represent the average value for each animal and per animal data points across sound-reward intervals are connected by the black dashed lines. B–E: Box plot showing the ratio of change in the number of baseline licks from control day (PBS or Saline) to manipulation day (MUS or CNO) across animals trained on the 1.5 s Delay and No-Delay tasks with muscimol infusions in the AC (B: N = 8, p = 0.121, Wilcoxon rank-sum test) and in the pStr (C: N = 5, p = 0.841, Wilcoxon rank-sum test), and with chemogenetic inactivation of the AC-pStr projections in the experimental group (D: N = 8, p = 0.295, Wilcoxon rank-sum test) and in the control group (E: N = 6, p = 0.3125 Wilcoxon rank-sum test). Lines connecting the circles represent the ratio of change in baseline licks for each animal when trained on the 1.5 s Delay and No-Delay tasks. In each box, the solid line indicates the median, and the bottom and top edges of the box indicate the 25th and 75th percentiles, respectively. The whiskers extend to the minimum and maximum values in the dataset. (TIF) [file pbio.3003209.s001.tif]

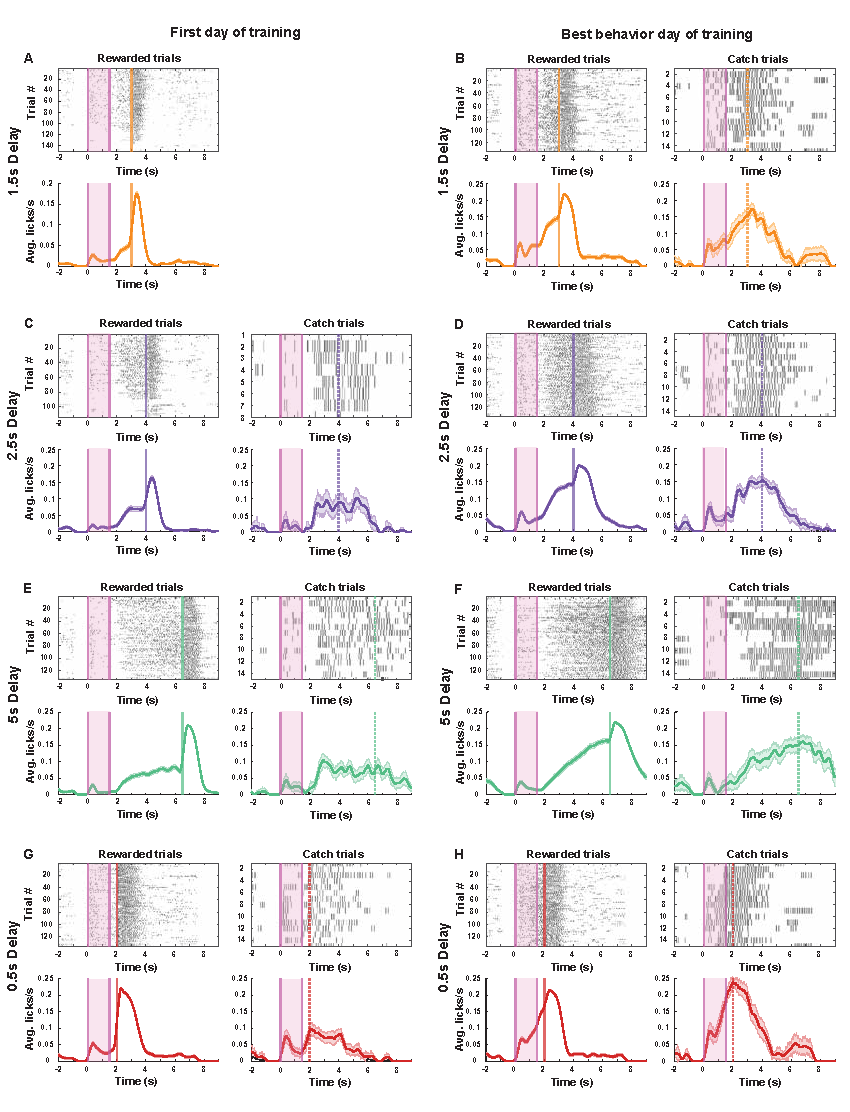

Supplement: S2 Fig — For each session, the four panels of plots show: Top: Peri-sound lick raster of the animal performing on rewarded (left) and catch (right) trials within the session. Bottom: Average peri-sound lick rate (licks/bin, bin = 1 ms) response curve (solid line denotes mean, shaded area represents SEM across trials) for the behavioral session above for rewarded (left) and catch (right) trials. Shaded pink region represents the 1.5 s long sound period. Solid and dotted lines represent when reward was given in rewarded trials and expected in catch trials. Black ticks represent licks. Each color represents the different sound-reward intervals. Note: The plots are shown in the order of training across sound-reward intervals, starting with the 1.5 s delay and ending with the 0.5 s delay. The very first session of training on the 1.5 s delay did not include catch trials. (PNG) [file pbio.3003209.s002.tif]

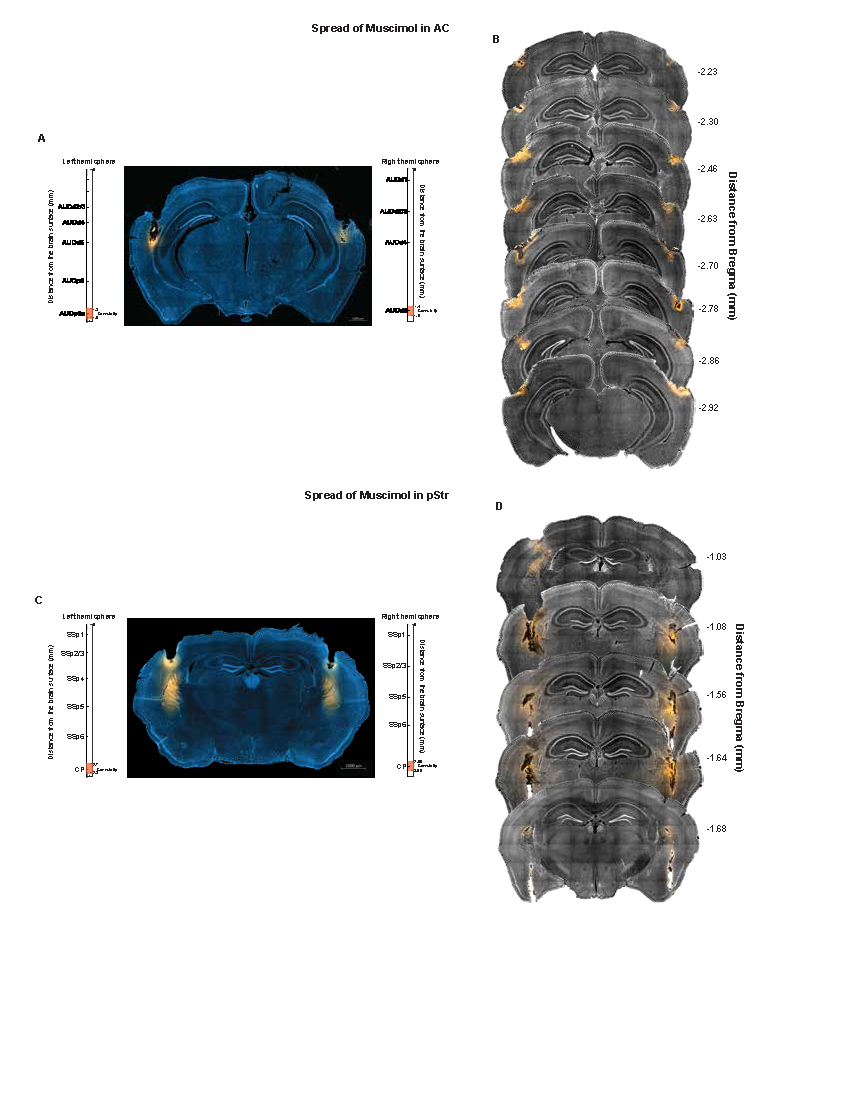

Supplement: S4 Fig — Brain slices acquired from an example animal with muscimol infusion sites seen in orange. Markers on either side indicate the depth at which muscimol was infused in the left and right hemispheres. Scale bar: 1000 μm. B. and D. Brain slices showing the spread of muscimol in the A/P axis for an example animal for AC and pStr. (TIF) [file pbio.3003209.s004.tif]

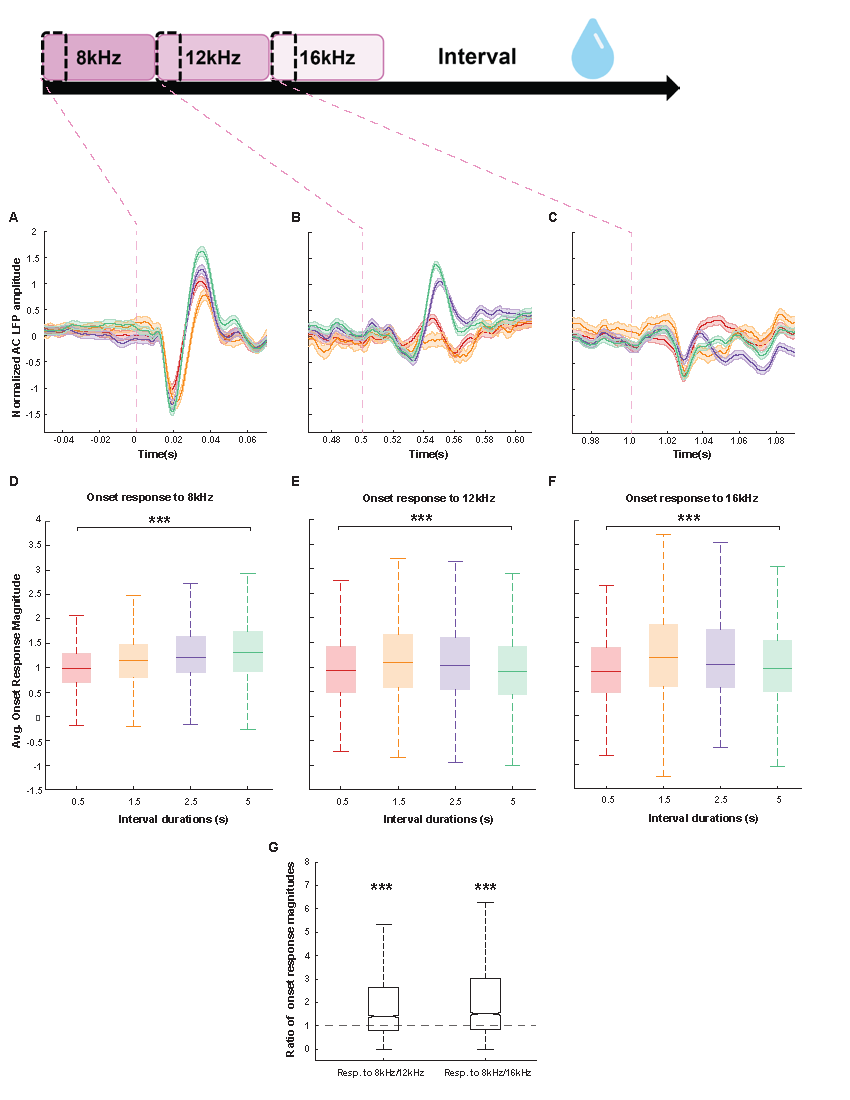

Supplement: S5 Fig — Dashed black lines represent onset of the tone as shown in the illustration above. D–F: Boxplot showing the AC LFP response magnitude computed across animals (N = 8) for each of the three tones across sound-reward intervals. In each box, the solid line indicates the median, and the bottom and top edges of the box indicate the 25th and 75th percentiles, respectively. The whiskers extend to the minimum and maximum values in the dataset. Comparison across sound-reward intervals yields – First tone: ***p = 1.42 × 10^−36^, Second tone: ***p = 1.11 × 10^−7^, Third tone: ***p = 4.38 × 10^−11^ (Kruskal–Wallis test). G. Ratio of the sound onset response magnitudes for (Left box) First to the second tone and (Right box) First to the third tone. (***p < 0.00001 compared against 1, Wilcoxon rank-sum test). (TIF) [file pbio.3003209.s005.tif]

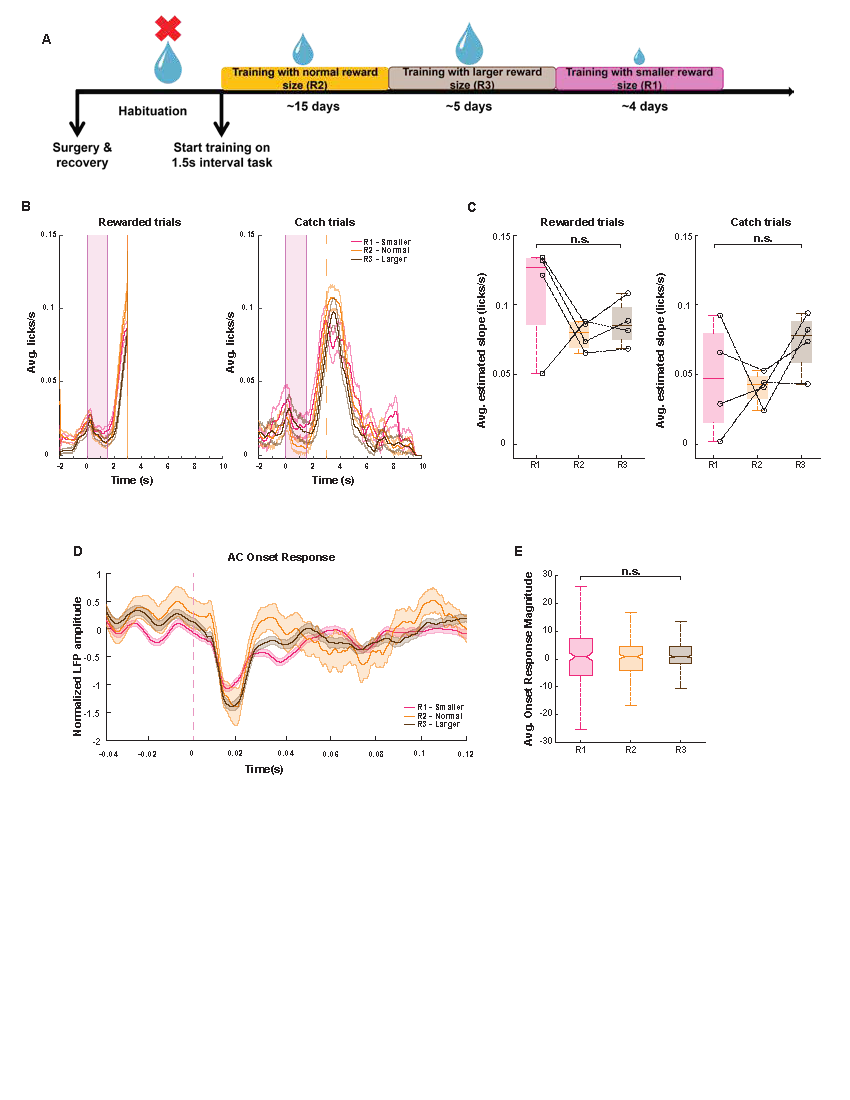

Supplement: S6 Fig — A. An Illustration of the behavioral timeline. B. Average peri-sound lick rate (licks/bin, bin = 1/20 ms) response curves (solid line denotes mean, shaded area represents SEM across trials) of an example animal trained to perform on the 1.5 s delay task with three different reward sizes represented by different colors. Left: Rewarded trials; Right: Catch trials. Shaded pink region represents the 1.5 s long sound period. Solid and dotted lines represent when reward was given in rewarded trials and expected in catch trials for each of the sound-reward interval. C. Box plot representing the estimated slope of predictive licking response curves for each of the three reward sizes across all animals (N = 4) for rewarded trials (left, p = 0.334, Kruskal–Wallis test) and for catch trials (right, p = 0.219, Kruskal–Wallis test). In each box, the solid line indicates the median, and the bottom and top edges of the box indicate the 25th and 75th percentiles, respectively. The whiskers extend to the minimum and maximum values in the dataset. Individual circles represent the average slope for each animal and per animal data points across sound-reward intervals are connected by the black dashed lines. D. Normalized average AC LFP responses to the sound onset from an example animal trained on the three different reward sizes represented by the different colors (solid line denotes mean, shaded area represents SEM across no-lick trials). Dashed pink line represents the period the sound onset. E. Boxplot showing the onset response magnitudes computed across animals (N = 4) for each of the reward sizes. In each box, the solid line indicates the median, and the bottom and top edges of the box indicate the 25th and 75th percentiles, respectively. The whiskers extend to the minimum and maximum values. (PNG) [file pbio.3003209.s006.tif]

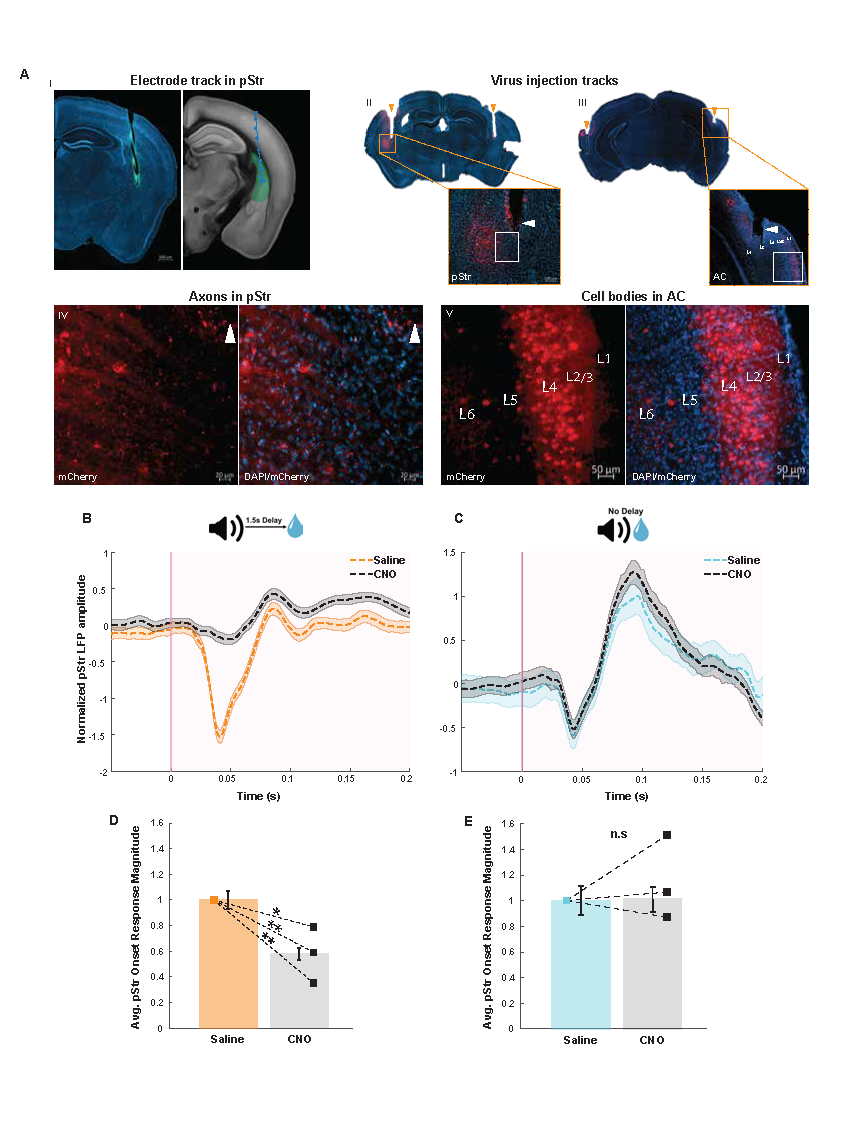

Supplement: S7 Fig — A. Histological validation of electrode position in pStr and chemogenetic virus expression in AC to pStr projections. I. Electrode position in pStr is denoted by the green dotted lines. II. and III. Representation of the virus injection tracks in AC and pStr. IV. and V. are magnified images from II and III showing axons in pStr and cell bodies in AC. B and C. Normalized average pStr LFP (solid dashed line denotes mean, shaded area represents SEM across no-lick trials) recorded in response to the sound onset from an example animal trained on the 1.5 s Delay task (B) and on the No-Delay task (D) with saline (orange/light blue) and CNO (black) injections. The shaded pink region represents the period from sound onset. D and E. Comparison of the normalized average pStr onset response magnitude computed across animals (N = 3) for the 1.5 s Delay task (C) and for the No-Delay task (E) on saline and CNO conditions. Error bars represent mean ± SEM across animals. Comparison between saline and CNO conditions yields *p < 0.05 for each animal when trained on the 1.5 s Delay task and was not significantly different when trained on the No-Delay task. (TIF) [file pbio.3003209.s007.tif]
