# Supplementary material for: An auditory cortical-striatal circuit supports sound-triggered timing to predict future events
Source: PLoS Biol. 2025 Jun 2;23(6):e3003209. doi: 10.1371/journal.pbio.3003209 (PMC12169527; doi:10.1371/journal.pbio.3003209)
Supplement: Attachment — Submitted filename: Rebuttal042125.pdf [file pbio.3003209.s008.pdf]

We thank the reviewers for their thoughtful and constructive comments, as well as their appreciation of our work. To address these comments we have conducted new experiments, performed additional analyses, and made edits and additions to the text and figures. We believe that our revised manuscript has substantially improved following these additions and edits. For convenience, the revised manuscript includes figures embedded within the text; however, we have also provided high-resolution versions of the figures as separate files. Below, we present a point-by-point response to the reviewers' comments.

REVIEWER 1

Reviewer #1: The present study explored the role of the auditory cortex and posterior tail of the dorsal striatum in the generation of sound-guided predictions. The paper is extremely well-written and easy to read. The experiments and analyses were generally well-designed and the results are clear. The findings represent an interesting and important contribution to our understanding of timing and learned auditory expectations. It is my hope that the comments below help the authors further strengthen what is already a solid paper.

We thank the reviewer for the appreciation of the work and its importance. We appreciate the constructive comments, which we have addressed point-by-point with clarifications, additions and changes to the manuscript text and figures, and new analyses.

Major:

1. The authors chose to chemogenetically silence pStr-projecting neurons in the auditory cortex at the level of the cell body. However, we know that at least some of these neurons send axon collaterals to multiple downstream targets, including the IC, MGB, and amygdala. Therefore, the authors cannot conclude that the monosynaptic projection from the AC to pStr is causally involved in the behavior or the associated evoked activity in the pStr— other pathway(s) might be responsible. The authors should either repeat the DREADD experiments, but use intracortical cannulae to directly silence the AC axon terminals in the pStr, thereby isolating the AC->pStr projection, or should walk back some of their claims and acknowledge this important caveat in their discussion.

While our approach ensured that only pStr-projecting AC neurons were inactivated, we agree that some of these neurons may send collateral projections to other targets. However, such collateral projections have been shown to be very sparse relative to the direct AC-Str projection (Li et al., 2021) (Fig. 6d-e) and previous studies have therefore used a similar dual-virus approach to target this pathway (Xiong et al., 2015; Znamenskiy & Zador, 2013). Moreover, our muscimol-induced inactivation experiments support the role of pStr itself in sound-triggered reward prediction. Nevertheless, as the reviewer notes, in the chemogenetic experiments we cannot fully rule out a contribution of the inactivation of collateral projections to the behavioral impairments. We therefore walked back some of the claims and acknowledge this point in the Results and added a section about it in the Discussion. Specifically:

1. *The section heading "Auditory cortical projections to posterior striatum are causally involved in sound-guided prediction of delayed reward" has been changed to "Activity of*

auditory cortical neurons that project to posterior striatum is necessary for sound-guided prediction of delayed reward"

2. *"we chemogenetically inactivated the projections from AC to pStr in animals trained to predict timed reward using a sound cue." Has been changed to "we chemogenetically inactivated pStr-projecting AC neurons and in animals trained to predict timed reward using a sound cue"*
3. *The sentence, "This approach allowed us to target the projections from the AC to pStr for chemogenetic inactivation" has been supplemented with "although sparse collateral projections may have also been targeted (Li et al., 2021) (see Discussion)".*
4. *The sentence "These findings further establish the causal involvement of the AC-pStr projections in animals' ability to predict delayed reward using a sound cue." has been replaced with "These findings further establish the causal involvement of pStr-projecting AC neurons in animals' ability to predict delayed reward using a sound cue."*
5. *In Discussion, we have added the following: "It should be noted that while our dual-virus targeting approach ensured that only AC-pStr projections were inactivated, some collateral axons from AC to other targets, such as the MGB, superior colliculus or dorsal periaqueductal gray, may have also been inactivated. Although these collaterals have been found to be sparse and therefore unlikely to dominate the behavioral outcome (Li et al., 2021), we cannot determine that the AC-pStr projection was the exclusive pathway mediating the behavioral impairment. Nevertheless, the anatomical robustness of the AC-pStr projection, as well as our findings that the inactivation of the AC-pStr pathway induced a significant decrease in the pStr response magnitude to the sound predicting the interval to reward, and that direct inactivation of pStr impaired the animal's ability to predict the sound-delayed reward time, suggest that the AC-pStr projection is involved in mediating this behavior."*

2. On a related note, the AC->pStr projection encompasses both excitatory and GABAergic inhibitory neurons. However, the authors used a pan-neuronal promoter (hSyn) to drive DREADD expression, which makes it difficult to interpret the downstream mechanisms involved. Do the authors believe the effects they observe are primarily mediated by silencing glutamatergic excitatory neurons? Or via disinhibition? A more detailed consideration of the possible mechanisms should be provided.

We agree with the reviewer and have added the following section to the Discussion that addresses this point:

"The AC projection to pStr encompasses both excitatory and GABAergic inhibitory neurons (Hunnicutt et al., 2016; Li et al., 2021; Rock et al., 2016). In this study, we cannot conclusively determine whether our findings are primarily mediated by silencing of glutamatergic excitatory neurons or via disinhibition. However, two pieces of evidence support the former alternative. We show that when the pStr-projecting AC neurons are chemogenetically inactivated, there is a significant decrease in pStr sound onset response magnitude (Supplementary Figure 6). Further, we show that a similar behavioral impairment as seen following the chemogenetic inactivation is observed following direct pharmacological inactivation of pStr using muscimol, a GABA-A agonist (Fig. 6). While further research is required to fully address this point, these findings suggest that the behavioral deficit we observed following chemogenetic inactivation is primarily mediated by silencing of excitatory neurons."

3. In Figure 5G, the authors report that in experimental animals, CNO treatment leads to a negative log PLR value for the no-delay condition. This suggests that CNO improves the ability of these animals to predict rewards that immediately follow the sound. How do the authors reconcile this observation with their working model?

We thank the reviewer for this observation, which we took a closer look at. We found that this trend resulted from two reasons. The first was a tendency for some animals in the no-delay conditions to slightly ramp up their licking in the few hundred milliseconds before the sound, apparently in anticipation of the simultaneous sound+reward (as licking in the pre-sound period is subtracted from the post-sound period, this contributed to a negative log-difference). In addition, two animals in this cohort did not increase their licking in response to the sound during catch trials in the PBS condition, indicating they had not formed the original association, and therefore the muscimol inactivation on these animals did not make sense and added "false" negative log PLR values to our population level data. As a result, we have made the following changes:

1. *To avoid pre-sound lick rampup artefacts, we have moved the baseline lick period back (while maintaining its duration) from just before sound onset (-750 ms to 0 ms), to just after the end of the preceding trials (-1900 ms to -1150 ms) for all calculations in the manuscript. We have indicated this change in the formula in the Methods section. The recalculation did not change any of the study's findings.*
2. *To be included in the analysis of the influence of neural inactivation on predictive licking, we now require that before inactivation, animals show significant predictive licking.*

   Applying the two changes described above, our log PLR value for the no-delay condition in chemo-expt group now reflects that at the population level, there is no significant effect ($p = 0.8483$, signrank when compared against 0) of CNO on the animals' ability to predict reward following the sound.

4. The analyses generally focused on the days that animals exhibited their best performance on the task. By excluding the other days, the authors are missing an opportunity to examine within-subject correlations between neural activity in the AC or pStr and behavioral performance. For example, one might predict that on days when the prediction does not appear to be very strong (as evidenced by little or inconsistent anticipatory licking), the LFP amplitudes would be weaker. Do the authors see any evidence of this?

We thank the reviewer for this suggestion. The reason we did not pursue this direction is that behavioral changes in performance were quite variable across individual sessions during the learning process. Nevertheless, to test these questions we performed a number of analyses (change in LFP across training, change in LFP vs. slope, change in LFP vs. trial-wise lick ramp time). Unfortunately, while some trends were noted, we did not find evidence for systematic relationships in our dataset (Reviewer Response Fig 1A-B). Thus, we were only able to establish a correlation between behavior and the LFP sound response magnitude when the animal is an expert on the task. However, to further establish the robustness of our findings and rule out the possibility that they are specific to the exact choice of best performance days, we reanalyzed the data using 4 alternative inclusion criteria (such as the last day of training [regardless of performance], the day before the best performance, and calculations of trial subsets). These analyses show that our findings hold true across a range of inclusion criteria (new Supplementary Fig. 3).

5. I was surprised to see that the authors did not cite or discuss a very relevant paper in their manuscript: Guo et al. (2019) Neuron 103:1164. Doing so would be helpful for placing their work in the context of the broader literature.

We thank the reviewer for bringing to our attention this oversight. We have included this relevant paper and discussed it in the Discussion section.

Minor

1. The authors claim that their sound stimuli were generated using a 25 kHz sampling rate. is this correct? If so, why was this sampling rate and/or the selected sound stimuli chosen? One of their sounds (16 kHz) falls above the system's Nyquist frequency, and was therefore likely was presented with significant distortions.

We thank the reviewer for noticing this error. The sound was generated at a sampling rate of 97.5 kHz (see the sound spectrogram in Reviewer Fig. 1E) and this has been corrected in the Methods section.

2.The histology images presented were not great.

We have edited and modified the resolution of the histology images to better reflect the targeting and manipulations done.

3. This is a very minor comment, but in some figures (e.g. Fig. 2, Fig. 6) the order of the figure panels is unconventional (i.e. panels A through D are presented in a clockwise manner). Readers (like myself) will likely look for the next panel by reading across the rows or down the columns. The authors may want to consider reorganizing the panels to accommodate these biases.

We have changed the order of the panels in all our figures to follow the order suggested by the reviewer.

4. At what dB SPL were sounds presented during the experiments?

The sounds were at 55-60dB SPL. This has been included in the Methods section.

REVIEWER 2

Reviewer #2 (Balazs Hangya): Suri et al. trained mice on a sound-cued delayed reward task and found that LFP responses recorded from the auditory cortex and the downstream posterior striatum represented the anticipated reward delay. Pharmacological inactivation of the auditory cortex or chemogenetic inactivation of their striatal projections impaired behavior. The Authors concluded that an auditory-striatal circuit supports timing prediction behaviors. The question is timely and the presented data are interesting. However, since only select data are featured leaving alternative conclusions open, a revision is needed before it can be judged whether the data indeed strongly support these conclusions.

We thank Dr. Hangya for the interest in the work and the appreciation of its timeliness. We appreciate the constructive comments, which we have addressed point-by-point with clarifications, additions and changes to the manuscript text and figures, new experiments, and new analyses.

Major points

1. One major shortcoming of the manuscript in the present form is that only selected parts of the data are presented. The figures show traces for the 'best behavior' day. Ideally, all sessions in example animals, traces averaged over all sessions within single animals, and grand averages over all animals should be shown to demonstrate consistency of the obtained data. Summary data are only presented in information-impoverished bar graph forms, where it is often not fully clear how the underlying data was generated. For instance, in Figure 1D, is this average of the best sessions of each animal, or average of the averages of all sessions from the animals? The former would mean most of the recordings not making it into the figures.

We thank the reviewer for this comment and the opportunity to clarify and expand the data represented in the figures. As our goal here is to understand reward time prediction and its underlying neurophysiological manifestations, we focused our analyses on the days when the animals show consistent and reproducible reward time-estimation behavior (via predictive licking). On earlier days of learning each sound-reward interval, animals did not display consistent behavior (as shown in new Supplementary Figure 2), limiting its informativeness in this context. However, following this comment we have made a number of changes and additions:

- *The data for Fig. 1D that the reviewer inquired about included slope values for each animal on their best behavior day per sound-reward interval and the bar plots showed the average across these slope values. Following the reviewer's comment, we have modified Fig 1D to a box plot and now include the individual data points.*
- *Further, to validate the robustness of our findings, we analyzed the data using 4 alternative inclusion criteria beyond the best-behavior day (such as the last day, the day prior, etc.). These analyses show that our findings hold true across a range of inclusion criteria, demonstrating their robustness (new Supplementary Figure 3).*

2. Relatedly, task intervals to calculate scalar measures (e.g. PLRs) sound somewhat ad hoc. What was the rational for choosing these variable interval boundaries? A demonstration that the results are robust to these choices could convince the readers that the actual choices of these somewhat arbitrary intervals do not affect the conclusions.

The lick time intervals for the calculation of the predictive lick ratio (PLR) was defined based on the animals' characteristic licking behavior. In Delay sessions (i.e., when there was a time delay between sound and reward, as in most of our data), well-trained animals typically ramped up their licking just before the anticipated reward time, and licked for a few hundred milliseconds. PLR was calculated exclusively on Catch trials, when no reward was delivered, so increased licking in this time window indicated anticipated reward. Specifically, we defined the lick window as going from -250 ms to +500 ms relative to anticipated reward time, as this 750 ms duration captured the typical period of enhanced licking in trained animals. Licking in this period is

compared (via subtraction) in the PLR calculation from licking in a baseline period, which precedes sound onset. This window was previously defined as -750 ms to 0 ms relative to sound onset, but following a different reviewer comment this window has been moved further back to start at -1900 ms, to avoid pre-sound increase in licking in the no-delay conditions (while remaining separate from the preceding trial). All results hold true following this change. For the No-Delay sessions, the predictive lick period is 0-750 ms relative to sound onset, and the baseline lick period is identical to the Delay sessions.

We have further clarified this in the Methods and provided examples of lick raster with the predictive lick periods and the baseline lick periods highlighted (Supplementary Figure 2).

3. An alternative conclusion would be that lick rates in the different conditions, as well as LFP responses represent subjective value of the cues or motivational value determining the vigor of behavioral response rather than interval timing: a delayed reward is of less value. This could be tested by an independent manipulation of reward value by changing reward size (and/or probability).

This is a very interesting point. To test this possibility, we conducted a new series of experiments, in which we manipulated the reward size while keeping the reward timing constant, as the reviewer suggested. Specifically, we trained a group of animals on the 1.5s Delay task with the original reward size and then instead of switching the sound-reward interval, we changed the reward size, first to a bigger reward size and then to a smaller reward size. We found that their post-learning behavioral performance (using slope), did not significantly differ across the three reward sizes (new Supplementary Figure 6). Further, the AC response magnitude to the sound onset did not significantly differ across these three reward sizes. As could be predicted from some previous studies (Hui et al., 2009; Rutkowski & Weinberger, 2005), there was a trend of increased response magnitude with increased reward size (Suppl. Fig.6). However, this trend is opposed to what would be predicted if the sound-reward delay was effectively reducing reward value, since in our main results we found that longer delays were associated with larger, rather than smaller responses. These results suggest that the AC LFP onset response magnitude to varying sound-reward intervals encodes the interval duration and not reward value. We have added these new data to the Results section.

4. Many lick patterns show some unexpected complexity, showing increased licking at sound onset, or maybe even before (?), then a decrease and a later sustained increase. Also, the baseline lick rate appears consistently above zero. However, these are hard to judge in the present figures; therefore, I suggest the inclusion of longer pre-stimulus intervals. Since animals were trained in blocks of days on the different conditions, does baseline licking show consistent variation according to the predicted interval? Because interval estimation only starts with cue onset, this would again strengthen a value-based interpretation.

We do observe that some animals tend to transiently lick immediately following sound onset, before showing the more sustained ramping pre-reward predictive licking. Interestingly, as noted in the next point, some animals showed an opposed brief cessation of licking in response to sound onset, before showing reward predictive licking. While these immediate modulations of licking to the sound onset could reflect a distinct and more reflexive response pattern compared

to reward-predictive licking, they were inconsistent and found in only a minority of animals and days, so we could not pursue this question within the scope of this study.

The animals generally did not show a significant increase before sound onset, although in a few sessions/animals in the no-delay condition we occasionally observed such a trend. Presumably, since in the no-delay condition sounds cannot be used to time delayed reward, some mice reverted to increase licking after some inter-trial interval duration. To avoid a contaminated contribution of this pre-sound licking, we have moved the 750 ms baseline lick window back to start at -1.9 s relative to sound onset (this is as far back as possible without overlapping the end of the preceding trials). This new baseline lick period was used for all analyses, and all our findings hold true after this change.

Accordingly, as the reviewer suggested, we now include a longer 2 s pre-stimulus interval for all our figures.

To address the final point, we tested whether the baseline lick rates differed across the sound-reward intervals. We found that baseline licking did not consistently or significantly vary according to the predicted interval ($p = 0.312$, Kruskal Wallis, new Supplementary Figure 1A).

5. Following up on this, the lick pattern in Fig.4B is hard to interpret: licking before stimulus but stop immediately after onset?

As mentioned above, we indeed occasionally observed a brief reduction in lick rate following sound onset, but as it was seen in only a minority of sessions/animals, and as it was temporally distinct from the reward-predictive licking, we did not pursue this further.

6. Relatedly, did any of the applied manipulations (and their respective controls) affect pre-stimulus lick rate?

We compared the average baseline licking between the control and manipulation days by computing the ratio of average baseline licking on control day to manipulation day, where the baseline lick period was defined as it was in the log PLR formula, to keep it consistent across all our computations. Our findings show no significant change in baseline licking following any of the manipulations (new Supplementary Figure 1B-E).

7. I do not see the systemic variation of AC LFP responses on the example trace in Fig.4D. This again points to the need to fill the data gap between a single session and the bar graph summaries.

To address the reviewer's comment, we have now included the trial-wise data, the unsmoothed version of the example provided in Fig 4 and a zoomed inset of the period over which response magnitude is computed. Please see the high-resolution figure image for better clarity.

8. The coordination analysis in Fig.7. is hard to interpret given that shuffled controls resulted in the same magnitude of cross-correlations. This may suggest that these correlations are stimulus-driven signal correlations and not trial-by-trial (noise) correlations; therefore, they do not reflect connections (unless I am truly missing something here). To show momentary coordination, trial-by-trial correlations beyond fluctuations of the mean (that is, mean-corrected or centered) that do not survive shuffled controls would be conclusive.

This is an important point which indicates we did not clarify the analysis properly. The shuffling of the cross-correlation in the original manuscript referred to random shuffling of the identity of the signals' brain regions- AC or pStr. The objective in this shuffling was to determine whether there is directionality in the cross-correlation, and it showed that while the peak magnitude of the cross-correlation did not differ when AC/pStr were shuffled (suggesting that its source is common input), the flanking sides of the cross-correlation did differ, such that in the true cross-correlation there was a significantly larger tendency for AC to precede activity in pStr by about 30ms than when the brain region identities were randomly shuffled. Importantly, in the original text we did not include "full shuffles", in which the AC/pStr traces were shuffled/jittered relative to each other. Following the reviewer's comment, we now include this shuffle, which results in a flat cross-correlogram as expected, and term it "Full Shuffling". For clarity, we term our original shuffle as "Directionality Shuffling".

9. According to leading theory, animals are expected to show noisy time keeping according to the subjective hazard model (see e.g. Janssen and Shadlen: A representation of the hazard rate of elapsed time in macaque area LIP, 2005). Do the Authors detect any indications of that?

While it would be very informative to relate our findings to the hazard rate model of elapsed time, we can unfortunately not do that within the current data for a few reasons. In the 2005 study, LIP neurons were shown to encode both elapsed time and the probability that the 'go' signal was about to occur. Critical for their analyses, 1. Spike times were recorded, allowing to quantify encoding at any time point, and 2. The trial wait times varied randomly across trials (drawn from various distributions). In our study, we recorded LFP activity rather than spikes and the trial durations were fixed in a given session (and typically for a few days). Thus, while our data was well-suited to test whether sound response magnitude changes with anticipated duration, they are not well suited to quantify of ongoing elapsed or anticipated time.

Minor points

1. Most details of the histology figures are not visible: the axes on the right, overlayed atlas images, scale bars.

We have edited and modified the resolution of the histology images to make them more legible.

2. Bar graphs should feature individual data points, with the chosen examples marked to demonstrate that they are rather typical than extreme ones.

We have replaced the bar plots with box plots and included individual data points to demonstrate the variability in our datasets.

3. When logPLRs are presented, the figure legend should clearly state the variables that went into the formula.

Done.

4. Number of training days for the no-delay task is missing in Fig.5A.

Done.

5. Improve the quality of the fluoromicrographs in Fig.6B.

Done.

6. Why is logPLR negative in Fig.5G, no-delay condition?

This is an important point that was also raised by Reviewer 1, major comment #3. Please see our response above.

7. CNO seems to have an effect in the control group as well in Fig.S2B and D. Please comment on this.

We compared the baseline corrected predictive licking for each of the three animals in the control group and find that two out of the three animals do not show a significant difference in predictive licking between saline and CNO days. However, since we only have 3 animals to report for this finding, we have decided to remove this supplementary figure from the manuscript.

8. Typo: Abstract line 36, animals' ability.

Addressed, thank you.

I believe that openness and transparency increase the fairness of peer review. Therefore, I am signing my reviews. Balazs Hangya

REVIEWER 3

Reviewer #3: Suri and colleagues describe behavioral, pharmacological, chemogenetic and neurophysiological studies in mice investigating the role that auditory cortex plays in predicting the timing of a sound-cued, delayed reward. Their main claim is that contrary to a traditional model of time perception that posits a "centralized clock", there is an auditory cortex-specific timing mechanism present at the onset of the sound that keeps track of the timing between a sound cue and reward. They devised a behavior for head-fixed mice to lick a water spout delivering reward at a fixed time after the onset of a sound cue, and claim that the rate of predictive licking ramps up in way that is commensurate with the latency to the expected reward time; that silencing auditory cortex or posterior striatum pharmacologically abolishes the predictive licking; that the relevant cue used by animals is the sound onset, which produces a local field potential (LFP) response whose amplitude scales with the duration of the delay, unlike the sound offset; that the projection from auditory cortex to the posterior striatum causally mediates the predictive licking; and that LFP coordination between these brain areas reflects the timing prediction.

Overall, this is a comprehensive set of experiments that tries to make an important mechanistic point about the representation of task-related time information, which has broad interest. The

auditory cortex neurophysiology and striatal projection necessity for a trace conditioning task are the particularly intriguing. The manuscript is generally written clearly, and the details are rigorous. Nevertheless, the interpretation is not fully convincing, and the authors should address the following major concerns.

We thank the reviewer for the appreciation of the work and its importance. We appreciate the constructive comments, which we have addressed point-by-point with clarifications, additions and changes to the manuscript text and figures, and new analyses.

1. The authors draw many of their behavioral conclusions by using AVERAGE measurements of the licking during the delay period, including the slope of the trial-AVERAGED rate of licking and the log ratio of the trial-average baseline-corrected number of licks between contrasting conditions. Averaging makes sense when the behavior across trials is more or less stationary. However, the example session shown in Figure 1B indicates that the lick rate is not increasing gradually within a trial to generate the predictive licking time course, but instead varies from trial to trial in the ONSET of when licking starts on individual trials - with a progressive adjustment over the course of a session towards refraining from licking until just after the reward is delivered. Thus, the averaged slope does not seem to be a rising prediction of when the reward will be given; rather it reflects a gradual adaptation across trials in a session so that the animal only has lick after the reward is actually presented. Have the authors analyzed licking in only the last several (rewarded or catch) trials of a session, and do those also show the same ramp up with a slope that scales with the interval duration?

While we agree that in the example lick raster shown in Fig. 1B there is a reduction in licking in the last trials, we believe that the slope of the trial-averaged lick rate is still a robust and reliable measure of reward timing expectancy for a number of reasons. First, while mild changes in lick rates and timing across a session can sometimes be observed, these are a minority of cases, as can be seen in the additional example rasters we now provide (Supplementary Figure 2). Second, while such differences in lick rates across trials could be observed for all sound-reward time intervals, they could not account for the finding that the slope varies systematically with reward timing. Third, all our key analyses are based on predictive licking in catch trials, where no reward is delivered, so if the animal had only licked following reward delivery as mentioned, we would not see differences in slopes in the catch trials (Fig. 1D, right). However, to validate these findings in the manner the reviewer suggested, we calculated the lick slope using only the last 30 trials per session and find that this slope also decreases as the sound-reward interval duration increases (new Supplementary Figure 3G), similar to our finding reported in Fig. 1D. Finally, we also applied the alternative manner of quantification the reviewer suggests in the next comment, which yielded similar results.

If the trial-averaged slope is not a good proxy for timing estimation on a trial-by-trial basis, have the authors tried estimating the onset of licking in a trial - for example by smoothing the discrete lick trace to find local peaks in the lick rate? From Fig 1C, it seems like the onset might shift with interval duration, but this could again be obscured by averaging across nonstationary trials.
If a reliable trial-by-trial estimate of timing prediction from the lick record is just not possible, then I would still suggest additional measures from the trial-averaged lick rate to bolster the evidence that the animals have a temporal sense of when the reward should be delivered. This

is especially true because there seem to be time points that do NOT correspond to the interval duration where the average lick rate starts to increase (most obvious in the 5s delay task in Suppl Fig 2. One possibility is in catch trials, it looks like the midpoint of the full-width half-maxima scales with the interval duration, and not just the precision, which suggest that the mice are aware of when the reward should have come and are not expending too much energy after the time point it was expecting. The midpoint might even be extractable on a trial-by-trial basis by smoothing the lick times in a trial, which could then be used to provide an additional estimate of timing prediction that could be compared across interval durations.

*We thank the reviewer for this interesting suggestion to bolster our behavioral analysis. In addition to calculating the average slope across rewarded and catch trials for each behavioral session, following the reviewer's suggestion we added an alternative lick rate measure. Specifically, for every trial, we defined the trial-wise lick ramp time by smoothing the lick trace and identifying the time point at which it crossed a threshold (mean + 2*std of average baseline licking for the session). We then averaged the trial-wise lick ramp times across trials (rewarded and catch trials) per session and compared these values across all the four sound-reward intervals. Consistent with our slope measure, we found that the average trial-wise lick ramp time increases as the sound-reward interval duration increases and is significantly different across the four intervals (Reviewer Response Figure 1F).*

2. The authors only use the "best" behavior days where the slope was the highest for the interval duration being trained. The quantitative assessments are then made based on comparing the slopes across interval duration. This seems like circular logic (see for example PMC2841687). Is there some other measure of behavior that can be used to restrict which sessions or trials are used to assess the prediction?

As the "best" behavior day for a given interval duration was chosen as the one with the maximum slope out of only sessions of that interval, and comparisons are then made between "best days" of different intervals, we do not believe there is a circular logic here. Nevertheless, to test and demonstrate the robustness of our findings to different choices of the behavior days, we analyzed the behavioral and neurophysiological data using 4 alternative inclusion criteria beyond the best-behavior day (such as the day before the best performance, the last day, etc.). These analyses show that our findings hold true across a range of inclusion criteria, demonstrating their robustness (new Supplementary Fig. 3).

3. The muscimol experiments (Fig 2 and 6) are very intriguing, but I have some methodological concerns. Water delivery is usually associated with a relay switch and valve that can produce audible noise or even vibration depending on where it is located; is that the case here, and is it also simulated during catch trials (just without water)?

We eliminated this risk by placing the valve outside our acoustically isolated behaviour box where the animals performed the task. Furthermore, our PLR calculations only include catch trials, in which there is no valve opening, so there can be no influence on behavior, and in cases where we do include rewarded trials, we only include data before reward delivery.

The actual water drop might also be visually apparent in the rewarded trials; the fact that there is often a rebound of licking around 4-5 sec after the reward is delivered (e.g., Fig 2B or 3B or 5D) also makes me wonder whether water might be slowly refilling the lick tube and becomes visually apparent, prompting further licking. Thus, it seems possible that the sharp, transient licking observed after sound onset in the no-delay experiments could be triggered by another sensory modality, which is why silencing auditory cortex has no effect - even if the animals were cortically deaf. The presence of a transient peak in the no-delay catch trials could be sufficient to counter that possibility and argue for intact sound processing of the cue, but I would expect some analysis and statement that there are no other sensory cues that might mediate reward delivery detection.

As mentioned above, our behavioral measures are based on either catch trials, where there is no reward, and therefore no potential other sensory cue (e.g., visual/olfactory) associated with it, or on the period before reward delivery in the rewarded trials. Therefore, while we agree that in principle animals might have been able to use visual or olfactory cues to time their licking in rewarded trials, such behavior would not be included in our measures. Specifically, in the no-delay conditions, our behavioral measure is only based on amount of licking in the catch trials, where there is no reward, and therefore also no auditory/visual/olfactory sensory cue associated with it. As such, only prior sound-reward association is expected to underlie licking. We therefore believe that the lack of influence of AC inactivation on licking in the no-delay condition shows that established no-delay sound-reward associations do not depend on the AC, and likely rely on subcortical structures.

Related, it is not clear from the text or figure (Fig 2D) whether the estimate of the log PLR is based only on the catch trials, rewarded trials or ALL the trials.

Log PLR was calculated only on the catch trials, and this is stated in the Methods section.

Furthermore, I found it odd that lick rates started increasing before the onset of the sound cue in some of these behavior figures (e.g. Fig 2C). If this is just an analysis artifact, perhaps the authors should avoid using smoothing that is noncausal, especially since the authors are trying to assess predictive behavior.

While there were occasionally minor increases (and decreases) in licking before sound onset, these were not systematic. To further demonstrate this, we provide additional examples of lick rasters in Supplementary Figure 2.

4. The muscimol experiments are informative, but there may be some alternative explanations that the authors should rule out. For one, rather than suppressing the ability of auditory cortex or posterior striatum to keep track of timing, perhaps the muscimol simply reduces overall motivation or drive, depressing overall lick rates. The Methods indicate that there was a wide range from 2-5 ml of water given to animals during behavioral training (Line 522) - was there any systematic difference in water drunk by animals on days they were given CNO vs PBS? Even if the motivation to drink overall was not different, and the ability to make a synchronous sound-water association was intact (Fig 2C, right; Fig 6C, right), can the authors rule out that muscimol to these areas was not simply reducing the motivation to lick in ANY trace cue-reward

paradigm without affecting the timing? For example, does the correlation (Fig 1D) between interval duration and lick slope (or other measure) go away with silencing? The addition of the 5 sec delay in Suppl Fig 2 for the chemogenetic experiment does not really answer this definitively. Indeed, it looks like there is predictive licking in Suppl Fig 2A under CNO, but just not to the same degree as for PBS.

The section in the Methods that the reviewer refers to indicates the range of amount of additional water given to each animal after behavioral training daily to keep them sufficiently hydrated. This variation was to compensate for individual animals' amount of licking during training. This has now been clarified in the Methods section. Nevertheless, to directly test whether the amount of overall lick rates was depressed by muscimol, we quantified the changes in baseline lick rate between control and manipulation days and found that there was no significant difference for all our manipulation experiments (new Supplementary Figure 1). We believe that these experiments, in addition to the animals' unimpaired licking to sounds associated with simultaneous reward (Fig. 2C), rule out the possibility that the manipulations simply reduced the motivation to drink. Indeed, we believe that the manipulations did not affect simultaneous sound-reward association but impaired predictive licking to rewards that were delivered following a time interval from the sound, indicates an impairment in sound-triggered prediction of time-delayed reward, which is what we argue for in these experiments. The reviewer is correct that in many cases predictive licking does not completely go away following the manipulations but rather that the amount before the anticipated reward is reduced. We believe this is consistent with an impairment in predicting delayed reward. To test whether the inactivations differentially impaired reward timing of varying intervals requires to carry out the inactivation experiments across all intervals, which is outside the scope of our study.

5. The conclusion that the mice use sound onset to predict reward timing should be caveated as it seems very likely that this is a task-specific finding. In particular, the task for Fig 3B was carried out in a block design where the offset of the sound was no more informative during training than the onset, and then during testing the offset was even less informative than the onset. Hence the animals only needed to use on the onset to predict in a block. While I would not require it, it would be interesting to know whether the use of a fixed interval (e.g. 1.5 sec) from the offset of a variable duration (.5 to 5 sec) sound within a training block would have produced a different result, since then referencing from sound offsets would have been critical for getting the reward at the right time. Without that though, unqualified statements such as in Lines 273-274 or Lines 431-433 about AC neural responses to sound onset encoding predicted time from sound to reward seem overstated. Related to Fig 3, it is not clear how the authors are measuring estimated slope and why it is so different between experiments (compare Fig 3C for a 1.5s delay to Fig 1D for a 1.5s delay).

We fully agree that the use of sound onset rather than sound offset is very likely task specific and have amended our statements in the references lines accordingly. Furthermore, we have added a section about this point to the discussion and included the proposed follow up experiment that the reviewer suggested.

Slopes in both the figures (Fig 3C vs. Fig 1D) are in the same range i.e. 10^{-4}. The difference is that 1.5s delay slope varies between 0.6 x 10^{-4} to 1.1x 10^{-4} in Fig 1D and goes all the way from 0.25x10^{-4} to 1 x 1^{0-4} in Fig 3C. These modest variations were due to the use of different cohorts of animals to address the different questions asked in these two figures.

6. The case made in Fig 4 is about onsets vs. offsets encoding predicted timing, but there is an implied correlation between the LFP peak amplitude at sound onset and the prediction of the reward timing based on lick rate. That is potentially quite exciting, but the evidence is not very strong as it is currently described. Did the authors run the actual correlation between these variables on a session basis across animals? Ultimately, it would be more convincing to be able to show at the trial level a correlation between the animal's behavioral prediction of the reward time and the magnitude of the LFP response. I understand that this might be noisy, but one could try looking at different quartiles of the data, blocking the trials into groups with different LFP magnitudes to see whether the average slope (or other trial-by-trial measure of timing prediction) scales accordingly.

The relationship we found between interval duration and LFP response magnitude is robust (systematically varies across >30% magnitude difference across interval durations) and highly significant. We fully agree that looking into this relationship at the trial level would be interesting, but as the reviewer noted, this is very susceptible to various sources of noise and trial-wise variations. To try to do this in the manner the reviewer suggested, we sorted the trials within each behavioral session by their AC LFP magnitudes, divided them into quartiles, and then computed the average trial-wise lick ramp time for each quartile. Across animals, we did not find a significant difference between quartiles (Reviewer Response Fig 1C).

Furthermore, it becomes clear in the Methods (Line 593) that the "sound" is actually a sequence of tones (any gaps?), which likely produces a distinct LFP response at each transition. Did any of those LFP responses correlate with the subsequent time to reward, or were those more like the offset response? Incidentally, it is never explained why the authors used such a sequence, and a brief comment about that at least in the Methods would be helpful. It is also unclear what normalization was performed and whether the y-axis has units - the Methods mention a baseline subtraction, but then the units should still be in microvolts.

We used a sequence of tones as a balance between acoustic simplicity, the prominence of time-varying sounds in ethological settings, and the known involvement of the AC in encoding such time-varying sounds, including via offset responses. This explanation has been added to the text.

There were no gaps between the tones beyond the 5 ms on/off ramps of each tone.

However, to address the reviewer's question, we quantified the magnitude of responses to the second and third tone and performed a similar analysis on them as for the first tone. We find that AC LFP responses to the second and the third tones in the sound sequence reduced were significantly smaller than to the first tone (new Supplementary Figure 5). We also compared the AC LFP response magnitudes to the second and third tones on the "best" behavior days across sound-reward intervals and see that while they are significantly different across interval durations, these response magnitudes did not increase with the interval duration as the response to the first tone. Rather, their responses tended to decrease with training, following the order in which these animals were trained on the four sound-reward intervals (1.5s → 2.5s → 5s → 0.5s) (Supplementary Figure 5). These results have been added to the manuscript.

All of our raw LFP signals were first filtered for movement artifacts and then z-scored within each session prior to further analysis, as described in the Methods (and therefore the units are no longer in microvolts). The baseline correction we refer to is done after z-scoring.

7. The intersectional chemogenetics can be quite powerful, and the data shown in Fig 5 is suggestive, but as in point #4 for Fig 6, silenced animals seem still able to predict the delay (location of the peak is similar), but just with overall reduced rates of licking. Specifically, Fig 5C seems to show that there is a rising lick rate during the delay period both during rewarded and catch trials, but that the overall number of licks is down in comparison to the saline trials. This can explain why there is an effect on log PLR even if animals are still able to predict the timing, which I suspect would show up in the slope.

To account for any differences in overall number of licks in CNO vs. Saline trials for each animal and its subsequent effect on our findings, we include the amount of licking in the baseline period in our log PLR calculations for all our chemogenetic and pharmacological manipulation experiments. Further, we show that there is no systematic difference in spontaneous/baseline licking between Saline and CNO days for across all experimental and control groups (Supplementary Figure 1D and E). As mentioned above, it is true that predictive licking is not abolished by CNO, but its baseline-corrected magnitude is significantly reduced.

Related, it is not clear why the no-delay trials have a negative log PLR, seemingly implying that silencing the auditory cortex to posterior striatum pathway made animals more likely to lick at the onset of the sound (example shown in Fig 5D appears to be the animal with the highest log PLR in Fig 5G). Is the mean/median log PLR for no-delay significantly different than 0? How do the authors interpret that?

This is an important point that was also raised by Reviewer 1, major comment #3. Please see our response above.

8. Fig 7 shows coordination between auditory cortex and posterior striatum, but does not make the connection to reward time prediction, other than being measured during the task. The trial-by-trial correlation shown by Fid 7D is interesting, but demonstrates the expected relationship for areas that are monosynaptically connected - although the delay of nearly 40 ms seems rather slow for such a connection. It's not clear that this connectivity serves anything specific about reward timing prediction, and could just be a part of executing the task, which would be evident even in the case of the no-delay task. The impact of this data for the reward timing prediction story would be more compelling if the authors could relate the brain area coordination and/or the posterior striatum neural activity to a trial-by-trial delay in licking. For example, in Lines 652-653, the authors mention that they did recordings in DREADD animals, but they did not show any positive control data on whether CNO attenuated posterior striatal activity and its coordination with auditory cortex.

We have taken a number of steps to address this comment. First, regarding the comment that "they did not show any positive control data on whether CNO attenuated posterior striatal activity": in our analysis of the data from the 3 animals in which we did LFP recordings while chemogenetically silencing the pStr, we found that in the 1.5s sound-reward delay conditions, CNO delivery significantly reduced pStr sound response magnitude (Supplementary Figure 7). Interestingly, the pStr sound response magnitude did not significantly change following CNO administration during the no-delay task. These results suggest that a key component of the sound responses in pStr is specific to delayed sound-reward conditions. A caveat of these results is that we only n=3 animals for this experiment, and it should therefore be treated with

caution. Indeed, for the same reason, our attempts at analyzing the influence of CNO administration on AC-pStr cross correlation yielded uninterpretable results. We therefore must conclude that we can indeed not determine with certainty that the coordination we observe between the AC and pStr is specific to reward time prediction. Instead, it may be a more general pattern of communication that can facilitate sound-triggered reward time prediction, but also other functions. We have added this important acknowledgment to the Discussion:

"Our LFP recordings in AC and pStr provided a quantification of the overall functional and temporal communication patterns across the AC and pStr. While we focused our analyses on the periods of sound-guided reward time prediction, we cannot determine that the coordination we observe between the AC and pStr is specific to reward time prediction. Instead, it may be a more general pattern of communication that can facilitate a range of functions. Future studies using single unit recordings could better elucidate how communication across these structures is manifested at the local ensemble level and across different periods and behaviors."

Related, in Fig 7B, how is the LFP normalized? If it is by subtracting the baseline, then why is .5 curve offset along the y-axis? This would seemingly affect the comparison of response magnitudes as a function of interval duration.

The representative LFP responses shown in Fig 4C, 4E and Fig 7B are not baseline-corrected. The only normalization performed on these responses is the z-scoring of the session-wise LFP signal in the pre-processing stages. We perform the baseline-correction while we compute the response magnitude. Both these points are described in the Methods section.

Other concerns

1. There is no quantitative assessment of the extend of virus expression or muscimol spread in auditory cortex of posterior striatum, and whether that correlated with stronger difference between PBS and MUS.

   We histologically verified the expression of virus and muscimol spread in the expected brains regions. We have added additional histology images to reflect the spread of muscimol in AC and pStr (Supplementary Figure 4). We used virus titers and muscimol volumes previously used in studies to achieve similar silencing.

2. Fig 1D: what are the units of slope along the y-axis? #/second? In other figures (e.g. Fig 2D), individual animals are shown as symbols - showing that here would also be informative.

   We have included the slope units in Fig. 1D and added individual animal data points to all our population-level plots.

3. Fig 2D and other log PLR plots: since the statistics used are non-parametric, the authors should consider showing the median and confidence intervals instead of mean and SEM. This seems particularly important for Fig 3D and 6D, where the means and error bars are misleading relative to the significance level of the tests applied.

We agree with the reviewer and have modified all our population-level plots to boxplots, indicating the median and the 25% percentile and 75% percentile lines.

4. Line 452 - ability to predict delayed reward time is not impaired - peak still occurs at the same place

   Following pharmacological inactivation of the auditory cortex using muscimol, we see that our animals can no longer predictively lick for reward at 1.5s (as shown in Fig 2B, catch trials and Fig 2D), while their motivation and ability to consume reward is intact, which is what the peak following reward delivery in rewarded trials shows (Fig 2B, rewarded trials).

5. Line 589: Provide the brand of speaker and its frequency range

   "4Ω, 3W Shutao magnetic speakers with a frequency range of 0-20 kHz" We have included this in the Methods section.

6. Line 608: "learnt" -> "learned"

   Done.

7. Line 632: what kind of connector was used?

   We used Ultraminiature PCB socket connectors (Mouser Part # 437-8618700810001101). This has been added to the Methods section.

8. Line 663-667: Why different measures for rewarded and catch trials?

   Please see our response for Reviewer 2, major comment 2.

9. Line 689: is there a typo in the equation? what was the window for no-delay?

   We have reformatted the equation to clearly indicate the predictive lick windows for delay and no-delay tasks separately.

10. Line 702-705: this seems like a confound - this is essentially the response during false negative trials, which presumably would not require animals to keep track of task timing. What is the justification?

    These sentences describe how we chose our "no-lick" trials for our LFP response analysis. We set this criterion to exclude the effect of licking on the sound onset-evoked LFP responses in AC and pStr, and to be able to purely derive the response to the sound only. Licking during the first 200ms from sound onset does not influence or provide evidence for animal's ability to predictively lick for reward, which is expected to occur much later.

11. Line 735: How many trials typically went into the cross correlation?

We quantified the number of trials that went into the cross correlated and have depicted it in Reviewer Response Fig 1D.

12. Supplemental fig 3: If this is normalized, why are the units still in uV?

The units were added by mistake. We have amended this in the current version of this figure.

Hui, G. K., Wong, K. L., Chavez, C. M., Leon, M. I., Robin, K. M., & Weinberger, N. M. (2009). Conditioned tone control of brain reward behavior produces highly specific representational gain in the primary auditory cortex. Neurobiol.Learn.Mem,.❺8(1), 27-34. https://doi.org/10.1016/j.nlm.2009.02.008

Hunnicutt, B. J., Jongbloets, B. C., Birdsong, W. T., Gertz, K. J., Zhong, H., & Mao, T. (2016). A comprehensive excitatory input map of the striatum reveals novel functional organization. Elife,.❶. https://doi.org/10.7554/eLife.19103

Li, Z., Wei, J. X., Zhang, G. W., Huang, J. J., Zingg, B., Wang, X., Tao, H. W., & Zhang, L. I. (2021). Corticostriatal control of defense behavior in mice induced by auditory looming cues. Nat. Commun,.78(1), 1040. https://doi.org/10.1038/s41467-021-21248-7

Rock, C., Zurita, H., Wilson, C., & Apicella, A. J. (2016). An inhibitory corticostriatal pathway. Elife,.❶. https://doi.org/10.7554/eLife.15890

Rutkowski, R. G., & Weinberger, N. M. (2005). Encoding of learned importance of sound by magnitude of representational area in primary auditory cortex. Proc.Natl.Acad.Sci.U.S.A,. 768(38), 13664-13669. https://doi.org/10.1073/pnas.0506838102

Xiong, Q., Znamenskiy, P., & Zador, A. M. (2015). Selective corticostriatal plasticity during acquisition of an auditory discrimination task. Nature,.❶87(7552), 348-351. https://doi.org/10.1038/nature14225

Znamenskiy, P., & Zador, A. M. (2013). Corticostriatal neurons in auditory cortex drive decisions during auditory discrimination. Nature,.0❺❸(7450), 482-485. https://doi.org/10.1038/nature12077
